# On the Treatment of Rotary-Lateral Curvature of the Spine*Read before the Medical Association, April 5th, 1881.

**Published:** 1881-06

**Authors:** Bernard Bartow


BUFFALO

MEDICAL AND SURGICAL JOURNAL.

VOL. XX.—JUNE, 1881.—No. 11.

### Original Communications.

### ON THE TREATMENT OF ROTARY-LATERAL CURVATURE OF THE SPINE.*

BY BERNARD BARTOW, M. D.

THE matter of spinal rotation, as it occurs among the phenomena of lateral curvature of the spine, is referred to generally by surgical writers as a change in the position of the vertebræ, (not necessarily connected with structural alterations) which is associated with the later stages of the disease; and in this connection attention is directed to it more as a complication than as a condition that has progressed, *pari passu*, with the other features of the disease. By all, its development is considered to add greatly to the difficulties of treatment, and to the restoration of the spinal column to its normal position.

The inference to be drawn from this is, that lateral inclination of the spine may exist as an abnormal condition without rotation of the vertebrae. Lateral curvature is also spoken of by writers as a condition in which purely lateral inclination of the column may be present independent of rotation of the vetebræ. This has always been regarded, however, as the condition

* Read before the Medical Association, April 5th, 1881.

representing the earliest stage of the disease. I believe the observation to be erroneous, that lateral curvature of the spine, unattended with structural changes in the vertebræ, occurs as a condition, purely, of lateral inclination. But, it seems to me, that in all instances of lateral curvature sufficiently developed to be recognized as such, there is rotation of the vertebræ, by which the normal curves are removed in part laterally to the right and left of the centre, simulating the appearance of a lateral bend in a part of the column; and that the degree of lateral incline is to a certain extent the measure of the rotation.

Rotation is a form of movement normally possessed by the spinal column. Gray describes it as being "produced by a twisting of the inter-vertebral substances; this, although only very slight between any two vertebræ, produces great extent of movement when it takes place in the whole length of the spine; the front of the column being turned to one or the other side. This movement takes place only to a slight extent in the neck, but is more free in the lower part of the dorsal and lumbar regions." Rotation of the spine is commonly seen as a normal movement in persons who habitually assume certain postures for the relief of the spinal muscles when fatigued. The most common of these resting positions is that known in military drill as "rest in place." In this and all analogous postures the muscles of the spine upon one side, and those of one lower extremity are relaxed, the spine and pelvis being supported by the ligaments, and the muscles of the other half of the body.

In this position of temporary muscular rest, spinal rotation occurs to a considerable extent; and in a person having a flexible spinal column, the rotation will closely simulate the appearance of lateral curvature of the spine. Importance is to be attached to this fact for the reason that, being a natural provision for the relief of muscular strain, persons possessing weak spinal muscles resort to it as a method of relieving the muscular fatigue, with such frequency as to cause the practice to be recognized as one exerting a strong influence upon the development of spinal mal-position. In this connection, also, in assuming postures such as tend to the development of spinal curves, persons always select those in which the muscular relaxation is the most complete; and with scarcely an exception, when the person is in the upright position, this relaxation of one set of spinal muscles is accompanied with spinal rotation. Lateral inclination at best is a constrained position and one not readily chosen for the purpose of procuring muscular rest.

The arrangement of the column being that of a series of curves, having their convexities directed antero-posteriorly, a force acting laterally upon these curves, instead of causing them to bend in a purely lateral direction, gives to the movement of the vertebræ a spiral course. This permits of lateral inclination by changing the direction of the principal curves, so that their convexities point diagonally as regards the central line of the body. This is invariably found to be the position of the vertebral curves in rotary lateral curvature. However slight may be the character of the change of spinal position, there will be found rotation corresponding to the extent to which the normal curves are removed laterally from the median line. The effect of vertebral rotation then—by giving a spiral direction to the axis of the spine—is to wheel the convexities of the spinal curves out of their median into a lateral position. The pelvis, not participating in the rotation, the relations of the trunk to the lower half of the body, are changed, so that the spinal curves project laterally, simulating a genuine lateral bend in the column. The lumbar and dorsal curves rotating in opposite directions, increase this apparent lateral inclination. The entire phenomena of lateral curvature, as it ordinarily occurs, can be accounted for by the act of spinal rotation alone. Were the conditions observed to be present with such regularity, due to any other movement than rotation, there would be as a consequence an order of symptoms, wholly different from those we are accustomed to deal with. Dr. A. B. Judson, in a paper upon "Rotary Lateral Curvature," read before N. Y. Academy of Medicine, says, "Rotation has been thought to occasion great difficulty in the mechanical treatment of lateral curvature. The treatment of lateral curvature is indeed beset with difficulty, not so much from the presence of rotation, however, as from the fact that the portion of the column which departs farthest from the normal position (the bodies of the vertebræ) cannot from their situation within the cavity of the trunk, receive direct mechanical support."

I have dwelt upon this matter of rotation chiefly with the endeavor to make myself clear in stating my belief, that the whole difficulty in the treatment of lateral curvature lies in the treatment of the rotation; that the treatment of lateral curvature consists in the treatment of spinal rotation; and that lateral curvature is spinal rotation due to unequal muscular action.

My attention has been fixed upon this matter by making plaster of Paris casts of the trunks in all my cases of spinal curvature. However indistinct the spinal rotation may have appeared in the living subject, it never failed to appear distinctly in the plaster casts; and in the examination and treatment of quite a large number of cases of all degrees of severity, I have never failed to find rotation the prominent feature.

A condition so universally present as a feature of lateral curvature seems to me to demand special attention in the mechanical treatment of the disease; and it is not saying too much to state that the unsuccessful results of treatment so commonly met with in this affection, are due more to the slight importance that has been attached to this feature of the disease than to any other that can be mentioned. In only a few of the multitude of appliances constructed for the relief of spinal deformity of this nature has there been an approach to correctness in the application of force for the twofold purpose of exerting spinal extension and opposing rotation. The apparatuses in which these properties have been combined most perfectly are of the nature of couches, upon which the patient reclines—extension and counter-extension being applied to the spine, while coincidently properly arranged levers act upon the thorax to oppose or overcome rotation. The various appliances designed to support the spine and at the same time allow the patient freedom from the evils of confinement, have been imperfect, more in the matter of exerting spinal extension than in any other respect—it being a well-known fact that spinal extension is necessary in connection with whatever force is employed, to overcome rotation, but that spinal extension alone is insufficient. Dr. Sayre has taught the principle by which spinal extension and fixation may be correctly maintained, by having the points of resistance in an apparatus applied to fixed points in the body. As he has stated, all appliances that exert an extending force against movable points in the body—e. g. the axillæ—are absurd in principle. The principal difficulty in the mechanical treatment of lateral curvature has been to establish a proper relation between the extending force and the force exerted to oppose rotation. Extension of the spine, as advised by Dr. Sayre, is insufficient to wholly overcome rotation, for the reason that the force is exerted upon the spine too near the centre of motion. The plaster of Paris jacket, in principle, maintains extension and fixation of the spine. By a repetition of this process of spinal extension and fixation, together with the influence of growth upon the spinal tissues, it is expected that the spine will eventually be restored to the normal position. But, unfortunately, this expectation is not realized in the greater proportion of instances. Extension persistently practiced, fails to correct rotation beyond a certain point, while such complete fixation of the spine, as the plaster jacket imposes, wholly removes the possibility of its taking place. The plaster brace maintains extension, but beyond the point where extension ceases to exert an influence upon rotation, the plaster of Paris brace has no further action.

Do we not possess the means for overcoming rotation as successfully as we are able to deal with the matter of spinal extension? I believe that we do, and that we can apply the force in one direction almost as easily as in the other. The ribs, from their attachment to the vertebræ, become indicators of spinal rotation. This is magnified in the ribs at points remote from the centre of motion. The rotation as exaggerated in the ribs produces the principal deformity of lateral curvature—the contour of the thorax becoming marked by depressions and prominences from participating in the vertebral movements. The ribs, then, may be used as levers by which force may be exerted upon the vertebræ to overcome rotation, provided that spinal extension is coincidently exerted. The plane of rotation being inclined, the direction given to the spine by these combined forces is spiral, and in a direction opposite to that in which the deformity has taken place.

In my attempts to make this idea practical, I have made a cast in plaster of Paris of the patient's trunk, using the plaster-jacket as a mould. The latter is applied while the patient is suspended, and the spine in a state of extension. The cast is then *treated* by cutting away the prominences and building out the depressions caused by the rotation, that exist at points, anatomically opposite. This should be done upon the entire thorax, and care should be taken to make the corrections in an order the reverse of that in which the changes have taken place. The changes, produced by rotation of the lumbar vertebræ, are to be treated in the same manner. The effect of this alteration is to restore to the plaster form the shape of the patient's trunk previous to deformity having taken place. From this corrected cast, a brace is made, by moulding upon it a piece of heavy belting leather that has been previously moistened in warm water. The leather, known as "unfinished" or "rough" leather, is preferable, as it has no oil in it, and has less of the leather odor, and when dry is much more firm and unyielding. The leather is moulded by winding it upon the cast with a light rope; it should be allowed to lap over in front, after the manner of a double-breasted coat; as this gives it additional firmness, where that is most needed. Before being removed from the cast, it may be perforated in every square inch of its surface with a ⅜-inch punch. When dry, the leather brace is sprung off the cast and allowed to dry further before being applied to the patient. When applied, it should be trimmed so as to exert no pressure in the axillæ or upon any prominent point liable to become easily chafed. The brace made in the manner I have described, being unyielding in character, is intended to exert constantly and gradually, and in the proper direction, a force that shall tend to make the yielding structures of the trunk adapt themselves to its form. Spinal extension is exerted upon the same principle as by the plaster of Paris brace, while in place of the fixation imposed by the plaster brace, there is a force constantly acting to resist rotation.

The pressure is brought to bear upon the points in the patient's trunk corresponding to those cut away on the cast; the surface over which the pressure is spread is so great as to cause no discomfort from any degree of force that can be exerted by the brace. In making the alterations upon the cast, the duration and degree of the curvature should guide us in the extent of the changes to be made. If the structures are rigid and unyielding, they will bear less pressure than if they are pliable and easily moulded. The amount cut away on the cast represents the special force the brace will exert upon the spinal rotation. Applied in this manner, the force is economized to the fullest extent, in that it is directed in the line of the spinal axis. The weight of the parts which the brace supports, is the measure of the force exerted upon rotation, which is acted upon so gradually as to give rise to no special discomfort.

Restoration of the column follows more quickly in the dorsal curve as a result of the more direct application of the force to that portion of the column. Rotation in the lumbar curve is less quickly overcome and does not wholly disappear until there is restoration of the muscular force which acts specially upon these vertebræ.

